# Concurrent Outbreaks of Hepatitis A, Invasive Meningococcal Disease, and Mpox, Florida, USA, 2021–2022

**DOI:** 10.3201/eid3004.231392

**Published:** 2024-04

**Authors:** Timothy J. Doyle, Megan Gumke, Danielle Stanek, Joshua Moore, Brian Buck, Timothy Locksmith, Kelly Tomson, Sarah Schmedes, George Churchwell, Shan Justin Hubsmith, Baskar Krishnamoorthy, Karalee Poschman, Brandi Danforth, Daniel Chacreton, the outbreak investigation team

**Affiliations:** Florida Department of Health, Tallahassee, Florida, USA (T.J. Doyle, M. Gumke, D. Stanek, J. Moore, B. Buck, T. Locksmith, K. Thomson, S. Schmedes, G. Churchwell, S.J. Hubsmith, B. Krishnamoorthy, K. Poschman, B. Danforth, D. Chacreton);; Centers for Disease Control and Prevention, Atlanta, Georgia, USA (T.J. Doyle, K. Poschman)

**Keywords:** hepatitis A, meningococcal disease, mpox, monkeypox, viruses, bacteria, sexually transmitted infections, meningitis/encephalitis, MSM, outbreaks, Florida, United States

## Abstract

In 2022, concurrent outbreaks of hepatitis A, invasive meningococcal disease (IMD), and mpox were identified in Florida, USA, primarily among men who have sex with men. The hepatitis A outbreak (153 cases) was associated with hepatitis A virus genotype IA. The IMD outbreak (44 cases) was associated with *Neisseria meningitidis* serogroup C, sequence type 11, clonal complex 11. The mpox outbreak in Florida (2,845 cases) was part of a global epidemic. The hepatitis A and IMD outbreaks were concentrated in Central Florida and peaked during March–­June, whereas mpox cases were more heavily concentrated in South Florida and had peak incidence in August. HIV infection was more common (52%) among mpox cases than among hepatitis A (21%) or IMD (34%) cases. Where feasible, vaccination against hepatitis A, meningococcal disease, and mpox should be encouraged among at-risk groups and offered along with program services that target those groups.

Outbreaks of hepatitis A have been previously reported among gay, bisexual, and other men who have sex with men (MSM) (*1*). Numerous outbreaks of hepatitis A among MSM were reported in Europe during 1997–2005 (*2*) and 2016–2017 (*3**–**5*). In the United States, hepatitis A outbreaks among MSM were reported in several locations during 2017–2018 (*6*,*7*). Outbreaks of invasive meningococcal disease (IMD) have also been previously reported among MSM (*8*–*10*). In late 2021, increases of hepatitis A and IMD were observed in Central Florida, primarily among MSM, prompting an investigation to guide the implementation of disease control measures.

During the period those 2 concurrent outbreaks were ongoing in Florida, a global epidemic of mpox emerged, in which sexual and intimate contact, particularly among MSM, was the primary mode of recognized transmission (*11*,*12*). In May 2022, a case of mpox associated with the global epidemic was identified in the United States. By November 30, 2022, the United States had >29,600 reported mpox cases and Florida had the fourth highest number of cases in the country (*13*).

We provide a descriptive, cross-sectional analysis of the concurrent outbreaks of hepatitis A and IMD in Florida in the context of an ongoing global mpox epidemic that also is disproportionally affecting MSM. Through this analysis, we attempted to identify common and distinct features of each outbreak and synergistic factors that might have affected disease progression and control.

## Methods

Hepatitis A and IMD are designated as reportable conditions in Florida; in 2022, mpox was reportable in Florida under the broader category of a disease of urgent public health importance (*14*). Cases for all 3 diseases are investigated by local staff of the Florida Department of Health (FDOH); investigations involve patient interviews to identify risk factors for illness and close contacts for possible prophylaxis or other needed follow-up actions. Interview questionnaires for each disease are standardized statewide; however, some questions differ between diseases. After recognition of an increased number of case reports, we created outbreak case definitions for hepatitis A and IMD (Table 1). We considered all mpox cases in Florida that met the national surveillance case definition to be outbreak related. The analysis period for this investigation included outbreak-associated cases for any of the 3 diseases that had illness onset during November 1, 2021­–November 30, 2022.

**Table 1:** Case definitions used in the investigation of concurrent outbreaks of hepatitis A, invasive meningococcal disease, and mpox, Florida, USA, 2021–2022*

Disease	Definition
Hepatitis A	
Inclusion criteria	Florida residents meeting the surveillance case definition for hepatitis A† with symptom onset during November 1, 2021–November 30, 2022
Confirmed case	A hepatitis A case with laboratory evidence of infection with HAV genotype IA, A17 cluster 21, or epidemiologically linked to a case with HAV A17 cluster 21 infection
Probable case	A hepatitis A case who identified as transgender or MSM or had sexual contact with MSM, irrespective of other risk factors for hepatitis A
Suspected case	A male hepatitis A case with unknown sexual history, and absence of other risk factors for hepatitis A (e.g., foreign travel, drug use, homelessness, incarceration)
Epidemiologically linked	Household or sexual contact during the 15–50 d before symptom onset
Exclusions	Cases with sequencing results indicating infection with HAV other than genotype IA, A17 cluster 21
Invasive meningococcal disease	
Inclusion criteria	Florida residents meeting the surveillance case definition for invasive meningococcal disease† with symptom onset during November 1, 2021–November 30, 2022
Confirmed case	An invasive meningococcal disease case who identifies as MSM or had sexual contact with MSM and evidence of serogroup C infection; or laboratory evidence of infection with ST11 CC11 serogroup C, with <100 SNPs difference to the outbreak strain, irrespective of sexual history
Probable case	An invasive meningococcal disease case who identifies as MSM or had sexual contact with MSM and for whom no additional serogroup, ST, or CC typing is available; or who had evidence of serogroup C infection regardless of sexual history
Exclusions	Cases with sequencing results indicating infection with *Neisseria meningitides* other than ST11 CC11 serogroup C or with >100 SNP differences to the outbreak strain
Mpox	
Inclusion criteria	Florida residents meeting the surveillance case definition for confirmed or probable mpox‡ with symptom onset during May 1­–November 30, 2022

*CC, clonal complex; HAV, hepatitis A virus; MSM, men who have sex with men; SNP, single-nucleotide polymorphism; ST, sequence type; STI, sexually transmitted infection. †Per Florida Department of Health definition (*15*). ‡Per Centers for Disease Control and Prevention definition (*16*).

### Hepatitis A Case Investigation

For all persons clinically diagnosed with hepatitis A, hospital and commercial laboratories were asked to forward available specimens to the FDOH Bureau of Public Health Laboratories (BPHL) for hepatitis A virus (HAV) genotyping based on the VP1-P2B junction region (*17*). Next-generation sequencing was performed on the MiSeq (Illumina) platform, and bioinformatics data processing used the Centers for Disease Control and Prevention (CDC) Global Hepatitis Outbreak Surveillance Technology (GHOST) platform (*18*). Investigation guidelines in Florida require that a case of hepatitis A in a food employee be further investigated to assess risk for possible food item or food preparation system contamination, which might also involve an environmental assessment. FDOH considered patron notification or suspension orders for food establishments, dependent upon the outcome of the risk assessments conducted.

### IMD Case Investigation

For persons with diagnosed IMD, isolates of *Neisseria meningitidis* were forwarded to BPHL for serogrouping by slide agglutination, and further characterization by whole-genome sequencing on MiSeq or NextSeq550 (Illumina) systems. Select specimens were also forwarded to CDC laboratories for additional characterization or confirmation. We analyzed sequencing data by using BPHL’s FLAQ-AMR pipeline (https://github.com/BPHL-Molecular/flaq_amr) to assess sequence quality and identify the sequence type (ST) and submitted data to CDC’s Bacterial Meningitis Genome Analysis Platform (BMGAP; https://github.com/CDCgov/BMGAP) to identify the clonal complex (CC) and to verify serogroup for each isolate (*19*). We identified outbreak-associated cases by serogroup, ST, and CC, and constructed phylogenetic trees.

### Mpox Case Investigation

In May 2022, FDOH notified healthcare providers and laboratories that mpox was considered reportable under existing rules. FDOH adopted the national surveillance case definition. That definition initially included clinical manifestations as a criterion, but on July 22, 2022, was modified to focus on laboratory results and epidemiologic risk factors for probable and confirmed cases (*20*).

Initially, all testing for orthopoxvirus and mpox virus infections was conducted at BPHL and CDC using previously described procedures (*21*,*22*). In July 2022, use of the nonvariola orthopoxvirus assay expanded to commercial laboratories (*23*). Molecular subtyping of isolates from mpox patients in Florida was not routinely done for surveillance purposes during the study period; however, CDC performed partial or full sequencing for a subset of samples (*24*,*25*).

### Epidemiology and Analyses

FDOH Bureau of Epidemiology operates an electronic patient-based reportable disease surveillance system known as Merlin. Using data from Merlin, we identified persons meeting the outbreak case definition for >1 disease in the 3 concurrent outbreaks. We used data from other surveillance systems operated by the Bureau of Communicable Diseases for sexually transmitted infections (STIs) and HIV to ascertain history and risk factors for reportable STIs (e.g., chlamydia, gonorrhea, or syphilis) and HIV infection. We matched patient profiles between systems by using name, sex, date of birth, and address information.

We calculated descriptive statistics to characterize outbreak-related cases by person, place, and time. We assessed pairwise differences in median age via Dunn test and conducted χ^2^ tests to compare cases of each disease by HIV infection, history of recent STI, and Orange County residency. We used 2-sided tests in all statistical analyses and considered p<0.05 statistically significant.

To assess the role of the public health response in controlling the concurrent outbreaks, we analyzed data from the statewide immunization registry by vaccine antigen and month of first dose administered. To distinguish outbreak response efforts from routine childhood or adolescent immunization, we limited data to vaccines administered to adults >18 years of age. We stratified data by persons receiving vaccine from a county health department (CHD) provider. To further characterize synergies in outbreak response efforts, we identified instances of vaccine administration to the same person, on the same day, against >1 of the diseases in the concurrent outbreaks. This activity was reviewed by the Ethics and Human Research Protection Program of FDOH and by CDC and was determined by both institutions to be public health practice, outbreak investigation, not requiring review and approval by an institutional review board.

## Results

### Hepatitis A Cases

During the analysis period, a total of 322 hepatitis A cases among Florida residents were reported to FDOH; 153 (48%) met the outbreak case definition. Of nonoutbreak cases during that period, ≈50% had an identifiable risk factor for hepatitis A, among which 60% were associated with recent international travel. Among the 153 outbreak-associated cases, 95% were in male persons and 5% in female persons, 74% were in MSM, and 21% were in persons with HIV; 1 death occurred (Table 2). Among persons for whom sexual history was obtained, 25% reported no sexual contacts in the previous 3 months; 8% reported >5 sexual partners, and 22% reported recent sexual contact with a person whose identity was not known. Seventeen cases were identified among food employees, but no foodborne transmission was documented. Environmental assessments performed at food establishments where exposure might have occurred did not necessitate FDOH orders for patron notification or suspension of service.

**Table 2:** Description of outbreak-related cases during concurrent outbreaks of hepatitis A, invasive meningococcal disease, and mpox, Florida, USA, 2021–2022*

Characteristics	Value		
	Hepatitis A	Invasive meningococcal disease	Mpox†
Total no.	153	44	2,845
Median age, y (range)	35 (9–75)	32 (8–77)	36 (0–81)
Race or ethnicity			
White	102/153 (67)	29/44 (66)	1,957/2,845 (69)
Hispanic	53/150 (35)	22/44 (50)	1,270/2,845 (45)
Sex			
M	145/153 (95)	36/44 (82)	2,786/2,845 (98)
F	8/153 (5)	8/44 (18)	59/2,845 (2)
Transgender	4/153 (3)	1/44 (2)	22/2,845 (1)
MSM‡	113/152 (74)	31/43 (72)	2,507/2,836 (88)
No. sexual partners during past 3 mo‡			
0	25/100 (25)	4/31 (13)	27/1,680 (2)§
1	44/100 (44)	17/31 (55)	914/1,680 (54)§
2–5	23/100 (23)	8/31 (26)	631/1,680 (38)§
>5	8/100 (8)	2/31 (6)	108/1,680 (6)§
Sex with unknown person in previous 3 mo‡	22/101 (22)	6/30 (20)	225/956 (23)§
HIV positive	32/153 (21)	14/44 (34)	1,473/2,836 (52)
Taking HIV preexposure prophylaxis	20/130 (15)	5/44 (11)	Unknown
STI in previous 2 y	42/153 (27)	9/44 (20)	1,567/2,836 (55)
Mpox virus infection in 2022	4	3	All
Recent international travel	12/153 (8)	1/44 (2)	199/2,845 (7)
Recent homelessness	8/153 (5)	2/44 (5)	25/928 (3)
Recent incarceration	1/153 (<1)	0/44 (0)	1/928 (<1)
Recent injection drug use	3/153 (2)	2/44 (5)	Unknown
Orange County resident	53/153 (35)	15/44 (34)	295/2,845 (10)
Food employee	17/121 (15)	7/44 (16)	Unknown
Hospitalized	119/153 (78)	41/44 (93)	162/2,845 (6)
Died	1/153 (<1)	9/44 (20)	3/2,845 (<1)
Outbreak case classification			
Confirmed	67/153 (44)	40/44 (91)	1,720/2,845 (60)
Probable	73/153 (48)	4/44 (9)	1,125/2,845 (40)
Suspect	13/153 (8)	NA	NA
Laboratory evidence of infection with outbreak genotype/sequence	60/153 (39)	34/44 (77)	NA

*Values are no. cases/no. reported (%) except as indicated. All risk factor questions relate to exposures in the 3 mo before symptom onset, except sexual history for mpox cases, which refers to the 3 weeks before symptom onset. Denominators vary based on available data for each variable. Percentages may not add to 100 due to rounding. MSM, men who have sex with men. †Outbreak ongoing, data through November 30, 2022. ‡Minors age <18 y excluded from numerator and denominator. §Number of sexual partners in 3 weeks before onset (mpox incubation period).

Among outbreak-associated cases, 67 (44%) met the confirmed case classification, among which 60 cases had matching HAV genotype IA, GHOST cluster A17 cluster 21 (a.k.a. SC076 US-Mexican); the remaining 7 confirmed cases had an epidemiologic link to a laboratory-confirmed case. We excluded 4 cases from the outbreak that otherwise met the probable or suspected outbreak case classification because HAV sequencing results did not match the outbreak cluster type. Of the 60 cases with the matching HAV outbreak cluster type, 2 patients appear to be outliers and had no identifiable risk factors or recognized exposures to HAV. One case was in a 74-year-old heterosexual female person and the other was in a 9-year-old male child. Of the 7 confirmed cases not genotyped but confirmed through epidemiologic linkage, 2 were linked to the 9-year-old patient, his parents, who later became ill, likely because of secondary household transmission; the remaining 5 cases were sexual or household contacts of persons who identified as MSM.

### IMD Cases

During the analysis period, 71 IMD cases among Florida residents were reported to FDOH. Of those, 44 (62%) cases were classified as outbreak associated. Among the outbreak cases, 72% were in persons who identified as MSM, 34% were in persons with HIV, and 20% of cases resulted in death (Table 2). The distribution of outbreak-associated cases by type of infection was 55% bacteremia, 20% meningitis, 5% septic arthritis, and 20% with >1 clinical syndrome. Case-fatality was highest (33%) among patients with bacteremia.

Among outbreak-associated cases, 40 (91%) met the confirmed case classification. Of those, 33 were caused by the ST11 CC11 outbreak strain; the other 7 confirmed cases were in persons who identified as MSM and had a serogroup C infection, but further strain characterization was not possible. Ten confirmed cases of the outbreak strain were in persons who did not identify as MSM, including a 77-year-old heterosexual woman with no known epidemiologic link to any other cases and 3 women who had no direct connection to one another but who were sexually active with the same male partner. Those 3 cases represent the only identified epidemiologic linkages among all the IMD cases in this outbreak.

### Mpox Cases

During May 10­–November 30, 2022, Florida reported 2,845 confirmed or probable mpox cases among residents. Most cases were locally acquired, but 14% of patients reported out-of-state travel during the 3-week incubation period. Most cases were among adult (>99%) and male (98%) persons. Among cases in adults, 88% were in persons who identified as MSM, but transmission through sexual contact between heterosexual persons was also identified. Among Florida cases, uncommon transmission routes were identified and have been previously reported by FDOH (*26*–*28*), including nonsexual household contact (n = 8) and occupational exposure among healthcare workers (n = 2).

Among mpox cases, 52% were in persons living with HIV and 55% in persons who had a history of >1 STI in the previous 2 years (Table 2). For the 1,414 cases with information available regarding previous HIV diagnoses, 10% were diagnosed within the previous year; 93% of cases were in persons who received HIV care within the previous year, 81% were in persons whose HIV infections were virally suppressed, and 6% were in persons who had a recent CD4 count of <200 cells/µL. Few (6%) cases were hospitalized because of mpox; 3 deaths occurred for which mpox was considered a causative or contributing factor. All 3 deaths were in persons who had a previous AIDS diagnosis, and 2 were unhoused.

CDC sequenced 17 mpox virus isolates and identified 16 as clade IIb subclade B.1 (*25*). One isolate collected early in the outbreak, from a patient with exposure in the United Arab Emirates, was identified as clade IIb subclade A.2 (*24*).

### Outbreak Overlap

**Figure 1:**
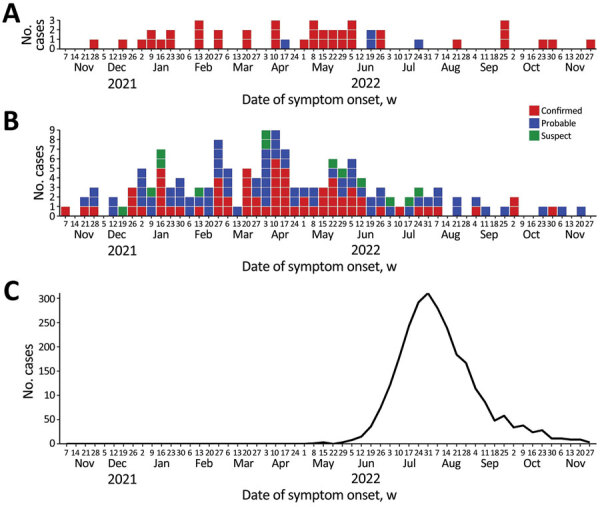
Epidemiologic curve of concurrent outbreaks of hepatitis A, invasive meningococcal disease, and mpox, by week of symptom onset, Florida, USA, November 1, 2021—November 30, 2022. A) Invasive meningococcal disease; B) hepatitis A; C) mpox. The case definition for invasive meningococcal disease cases had 2 categories: confirmed and probable. The case definition for hepatitis A cases had 3 categories: confirmed, probable, and suspect. The graph for mpox includes all confirmed and probable cases.

We observed temporal overlap for the 3 outbreaks; hepatitis A cases occurred somewhat earlier, and peak incidence was during late March and early April 2022 (Figure 1). Peak incidence for IMD occurred a few weeks later, and 13 cases were reported over a 6-week period during May–June. The number of hepatitis A and IMD cases declined as mpox cases rapidly increased; mpox incidence peaked during late July to early August, then rapidly declined. We did not identify any instances of the same person being part of both the hepatitis A and IMD outbreaks. However, among mpox cases, 4 patients were also part of the hepatitis A outbreak, and 3 others were part of the IMD outbreak (Appendix Table 1).

**Figure 2:**
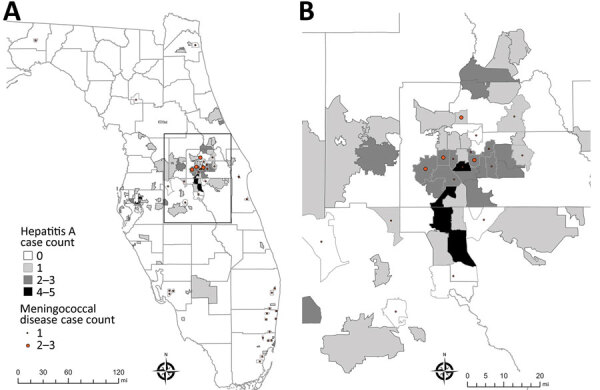
Spatial distribution of outbreak-associated cases of hepatitis A and invasive meningococcal disease in an investigation of concurrent outbreaks of hepatitis A, invasive meningococcal disease, and mpox, Florida, USA, 2021–2022. A) Locations of cases by postal (ZIP) code of patient residence; B) detail of box from central Florida in panel A, in which >1 case of each disease were reported in the same postal code. Outside the area represented in that panel, no cases of both diseases were identified in the same postal code. Invasive meningococcal disease cases were georeferenced by postal code centroid. Small polygons are outlines of postal code areas. Light gray lines indicat county borders; dark gray lines indicate postal areas where instances were identified.

The greatest concentration of outbreak-associated hepatitis A and IMD cases occurred in the central region of Florida (Figure 2). Orange County had the highest number of cases for both diseases: 53 (35%) cases of hepatitis A and 15 (34%) cases of IMD. On the basis of patient residence, we identified 12 postal (ZIP) codes with >1 case of both hepatitis A and IMD, 3 of which had >2 cases of each disease (Figure 2). For hepatitis A, several cases occurred in west-central Florida (Hillsborough and Pinellas Counties), where IMD was not observed. Several IMD cases occurred in southeastern Florida (Miami-Dade, Broward, and Palm Beach Counties), where outbreak-associated hepatitis A was not frequently observed.

**Figure 3:**
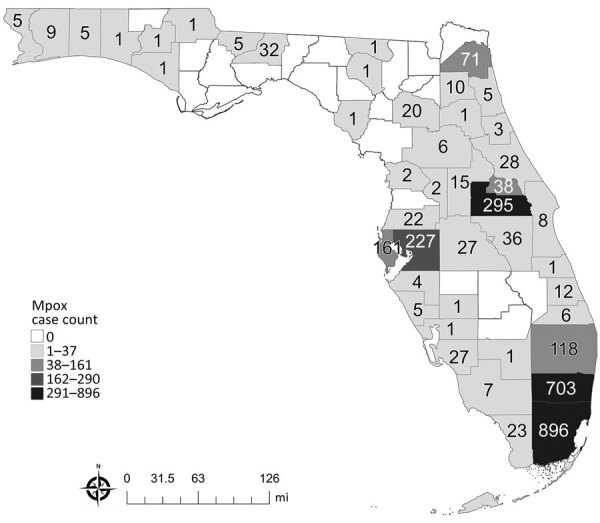
Distribution of mpox cases by county of residence in an investigation of concurrent outbreaks of hepatitis A, invasive meningococcal disease, and mpox, Florida, USA, 2021–2022. Numbers on map indicate numbers of persons with mpox reported to Florida Department of Health, by the person’s county of residence.

Mpox cases in Florida were more widely dispersed; cases were reported in 45 of 67 counties. The highest concentration was observed in the southeast region, and 56% of all cases were reported in Broward and Miami-Dade Counties (Figure 3). Orange County reported the third highest (295; 10%) number of mpox cases. The 7 most populated counties in Florida accounted for 87% of mpox cases reported in the state. Exposure occurred exclusively outside the United States for 5% of Florida mpox cases, and an additional 2% of cases reported multiple exposure locations that included >1 location outside the United States.

Comparing cases by disease, the median age of IMD case-patients was 32 years, which was significantly younger than for hepatitis A (35 years; p = 0.0145) and mpox (36 years; p = 0.0043) case-patients. We detected statistically significant differences when comparing cases of each disease by HIV infection, history of recent STI, and Orange County residence (p<0.0001). HIV infection was more prevalent among mpox (52%) cases than among IMD (34%) or hepatitis A (21%) cases. In addition, STI in the previous 2 years was more prevalent among mpox (55%) cases than among hepatitis A (27%) or IMD (20%) cases. A lower percentage (10%) of mpox cases were among Orange County residents than were IMD (34%) and hepatitis A (35%) cases.

### Control Measures

**Figure 4:**
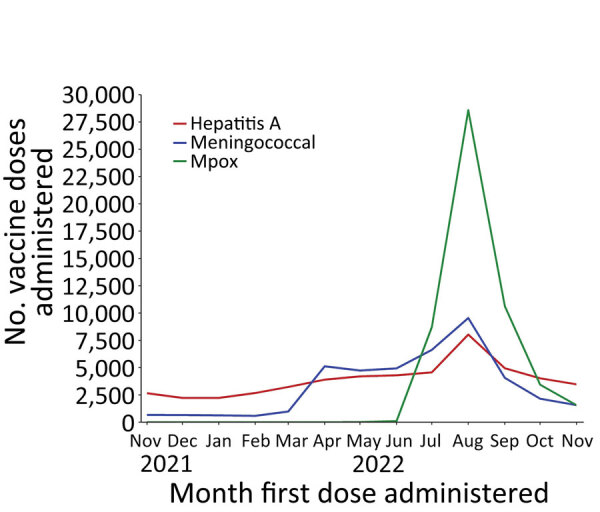
Persons vaccinated by month and antigen during concurrent outbreaks of hepatitis A, invasive meningococcal disease, and mpox, Florida, USA, 2021–2022. The figure shows the number of adult persons (>18 years of age) vaccinated with their first dose, by month the first dose was administered. For hepatitis A, the number includes persons vaccinated with any Food and Drug Administration (FDA)–approved vaccine against hepatitis A virus. For meningococcal disease, the number includes persons vaccinated with any FDA-approved serogroup ACWY vaccine (Menveo [GlaxoSmithKline], Menactra [Sanofi Pasteur, Inc.], MenQuadfi [Sanofi Pasteur, Inc.]). For mpox, the number includes persons vaccinated with JYNNEOS (Bavarian Nordic). FDA, Food and Drug Administration.

JYNNEOS (Bavarian Nordic) vaccine for preventing mpox became available in limited supplies in Florida in late May and June 2022. As the vaccine supply increased, persons vaccinated with their first dose increased markedly; in July and August, >53,000 adults in Florida received >1 dose (Appendix Table 2). Immunization against hepatitis A and IMD also increased markedly among adults during that timeframe (Figure 4). In August 2022, of the 28,592 persons receiving a first dose of JYNNEOS vaccine, 7,305 (26%) received >1 other vaccine against either hepatitis A or IMD on the same day; 2,979 (10%) received vaccines against all 3 diseases on the same day. More than half of adult immunizations for hepatitis A and IMD in August were administered by CHD providers. In contrast, during months outside the August–October period, <38% of adults receiving a vaccine for either disease received that dose from a CHD provider.

The hepatitis A and IMD outbreaks concluded by the end of 2022, but mpox cases continued to be reported in Florida as of January 2024. By that time, a total of 2,957 mpox cases had been reported since the start of the outbreak, which is considered ongoing.

## Discussion

Outbreaks of hepatitis A and IMD among MSM were ongoing in Florida when the emerging mpox outbreak added further complexity and urgency to the public health response. In addition, the introduction of mpox might have hastened the decline of cases in the hepatitis A and IMD outbreaks, possibly due to multiple factors. First, the negative perceptions and fear of acquiring mpox might have led to changes in sexual behaviors among MSM, particularly persons outside of monogamous relationships. In a national survey of 703 MSM and transgender women conducted in August 2022, more than half reported a change in their sexual behavior because of the mpox outbreak; 40% reported limiting the number of sexual partners, and 24% avoided having any type of sex (*29*). Similarly, in another survey of MSM conducted in August 2022, a total of 50% of respondents reported reducing 1-time sexual encounters and 50% reported reducing sex with partners met on dating apps or at sex venues (*30*).

The response to mpox might also have hastened the end of the hepatitis A and IMD outbreaks through coordinated immunization outreach efforts targeting overlapping risk groups. Before August 2022, demand for JYNNEOS vaccine was strong, but supplies were limited (*31*,*32*). As the vaccine became more readily available (*33*), CHDs in Florida conducted outreach activities to provide mpox vaccination to gay, bisexual, and other MSM, which also helped provide vaccination against hepatitis A or IMD to the same persons, where indicated (*34*,*35*). The strong interest and demand for JYNNEOS vaccine among MSM likely led some persons to get vaccinated against hepatitis A and IMD who might not otherwise have been vaccinated. Vaccines administered by CHDs were provided free of charge to the client and, in some counties, made available outside nightclubs and at social venues or community events. That strategy leveraged lessons learned from HIV prevention programs, including making services available to persons who would most benefit, where they most likely would be, and when they would most likely be comfortable (*36*).

We observed a high percentage of concurrent HIV infection among hepatitis A, IMD, and most notably, mpox case-patients. We observed a similar pattern regarding history of an STI in the previous 2 years. The duration of HAV viremia and stool shedding might be longer in persons with HIV (*37*), and persons with HIV who have low CD4 cell counts are also more susceptible to invasive disease when exposed to *N. meningitidis* (*8*,*10*). Some researchers have proposed the concept of syndemics to refer to multiple diseases occurring in the same population that have a synergistic effect on the progression or outcome of each disease (*38*,*39*). HIV and STIs are frequently cited examples of infectious disease syndemics. We observed current or recent infections in the same person, including HIV, >1 STI, mpox, and hepatitis A or IMD, in a setting of concurrent outbreaks. A single case report involving a similar combination with severe hepatitis A was previously reported (*40*).

Of note, 25% of adult hepatitis A case-patients in the outbreak reported no sexual activity in the previous 3 months. That might be the result of unreliable information regarding sexual history provided by case-patients. However, it also might be explained by possible undetected spillover into foodborne transmission or environmental surface contamination at venues that cater to an exclusively or predominantly lesbian, gay, bisexual, and transgender clientele, a possibility that has been noted in similar outbreaks (*41*). No common venues were identified in our outbreaks but interviewed case-patients frequently declined to identify specific venues. Moreover, some confirmed cases in the hepatitis A outbreak in persons who were not sexually active, such as the elderly woman and young boy, might be explained by limitations of HAV subgenomic sequencing to detect variations in phylogenetic relationships (*42*).

Simultaneous response to these 3 concurrent outbreaks highlighted differences in public health surveillance, investigation, and response activities for diseases considered sexually transmitted compared with other communicable diseases, such as hepatitis A or IMD, that have multiple transmission modes that also can include sexual contact. Many US public health departments, including FDOH, are organizationally separated between general communicable diseases and control programs related to STIs and HIV. Barriers often exist for integration of staff and surveillance information resources across those program areas. Nevertheless, we found added value in close collaboration and integration of case-related data in response to these concurrent outbreaks. Further integration across program areas could provide added benefit in disease control efforts, particularly those involving overlapping risk groups.

When the global mpox epidemic emerged in 2022, and transmission among MSM was quickly recognized, investigation methods in the United States (e.g., questionnaires and case interviews) rapidly aligned to methods typically used for STIs and HIV (*36*). In Florida, methods included using experienced disease intervention specialists (DISs) who have advanced skills in eliciting sensitive details regarding sexual history. In contrast, case investigations for most reportable diseases in Florida, including hepatitis A and IMD, do not routinely include detailed information on gender identity, sexual orientation, or sexual history, nor are those investigations routinely conducted by trained DIS staff with specialized interviewing skills. Thus, data collected during these concurrent outbreak investigations were not standardized nor consistently gathered across diseases, limiting direct comparisons. Furthermore, we did not directly compare MSM status across the diseases because MSM was a criterion within the outbreak case definitions for hepatitis A and IMD but not for mpox. Nor did we compare race or ethnicity across the 3 diseases because population demographics differ by region in Florida and mpox was much more heavily concentrated in southern Florida than the other 2 diseases.

This investigation was also limited by an inability in many instances to identify close contacts, particularly known or anonymous sexual contacts. Thus, epidemiologic linkages between cases were often difficult or impossible to establish. The lack of available specimens from all patients also limited efforts to fully use molecular and genomic analyses to identify connections between cases or to rule out probable or suspected cases that might not have been truly related to the hepatitis A or IMD outbreaks.

Recognition of factors associated with an emerging syndemic early in its course facilitated improved data sharing and leveraged limited resources among public health programs responsible for disease control in Florida. In addition, combined vaccination efforts targeting overlapping at-risk populations likely contributed to the control of these outbreaks.

In conclusion, we found that cross-training between DIS and general communicable disease surveillance investigators might improve the quality of information collected through case interviews, particularly information related to sexual history. Disease prevention opportunities, also provided by DIS, might be enhanced through an integrated approach to screening, education, and linkage to care for multiple infectious diseases across program areas. Finally, vaccination against hepatitis A, meningococcal disease, and mpox should be encouraged among MSM, consistent with national guidelines (*33*–*35*) and, where feasible, offered with other program services to the same at-risk population.

Appendix Additional information on concurrent outbreaks of hepatitis A, invasive meningococcal disease, and mpox, Florida, USA, 2021–2022.
