# Supplementary material for: Concurrent Outbreaks of Hepatitis A, Invasive Meningococcal Disease, and Mpox, Florida, USA, 2021–2022
Source: Emerg Infect Dis. 2024 Apr;30(4):633–43. doi: 10.3201/eid3004.231392 (PMC10977815; doi:10.3201/eid3004.231392)
Supplement: Appendix — Additional information on concurrent outbreaks of hepatitis A, invasive meningococcal disease, and mpox, Florida, USA, 2021–2022. [file 23-1392-Techapp-s1.pdf]

Article DOI: https://doi.org/10.3201/eid3004.231392

EID cannot ensure accessibility for supplementary materials supplied by authors. Readers who have difficulty accessing supplementary content should contact the authors for assistance.

Concurrent Outbreaks of Hepatitis A, Invasive Meningococcal Disease, and Mpox, Florida, USA, 2021–2022

Appendix

Appendix Table 1. Characteristics of mpox cases also part of the hepatitis A or invasive meningococcal disease outbreaks, Florida, 2022*

Mpox onset, mo.	Other disease, onset month	Age, y	Race/ Ethnicity	Gender†	Sexual Orientation	HIV+	CD4 count, cells/µL‡	Lab findings	Note
Jul	IMD, meningitis, Jan	47	B/NH	M	Lesbian or Gay	Yes	61	Serogroup C	Denies recent sexual activity
Aug	IMD, meningitis, Feb	30	B/NH	M	Bisexual	Yes	19	ST11 CC11	Reported 2–5 sexual partners; 4 STI in past 2 y; died 2022 Nov
Oct	IMD, meningitis and bacteremia, Jul	31	B/NH	M	Bisexual	Yes	39	ST11 CC11	1 STI past 2 y; history of cancer
Aug	HepA, Jan	29	W/H	Transgender M-F	Other	Yes	572	Not genotyped	2 STI past 2 y; reports 1 male partner
Aug	HepA, Jun	33	W/H	Transgender M-F	Lesbian or Gay	No, on PrEP	Not available	Not genotyped	4 STI past 2 y; reports 1 male partner
Aug	HepA, Oct	31	W/H	M	Lesbian or Gay	Yes	889	Not genotyped	Recent syphilis, 2 STI past 2 y; reports 2–5 male partners
Aug	HepA, Nov	31	W/H	M	Lesbian or Gay	Yes	299	Not genotyped	Recent syphilis, 5 STI past 2 y; reports >5 male partners

*B, Black; CC, clonal complex; H, Hispanic; HepA, hepatitis A; IMD, invasive meningococcal disease; NH, non-Hispanic; PrEP, pre-exposure prophylaxis for HIV infection; ST, sequence type; STI, sexually transmitted infection; W, white.
†Gender same as birth sex, except where noted.
‡All reported CD4 cell counts measured during September–December 2022.

Appendix Table 2. Adults vaccinated in Florida, by antigen and month, during the outbreak November 2021—November 2022*

		Vaccinations, no. (% by CHD)					
Year	Month	Hepatitis A†	Meningococcal‡	Mpox§	Hepatitis A & meningococcal¶	Mpox & hepatitis A or meningococcal#	Mpox & hepatitis A & meningococcal**
2021	Nov	2,654 (20.2)	666 (16.7)	1 (0.0)	147 (32.7)	0	0
2021	Dec	2,225 (21.7)	658 (18.8)	1 (0.0)	145 (39.3)	0	0
2022	Jan	2,229 (20.7)	627 (20.4)	3 (0.0)	150 (47.3)	0	0
2022	Feb	2,681 (29.7)	589 (22.6)	1 (0.0)	173 (35.3)	0	0
2022	Ma	3,233 (26.0)	987 (26.2)	0	254 (50.4)	0	0
2022	Apr	3,899 (24.5)	5,124 (13.3)	1 (0.0)	478 (45.6)	1 (0.0)	0
2022	May	4,202 (25.4)	4,738 (19.4)	8 (100.0)	645 (52.1)	0	0
2022	Jun	4,295 (25.3)	4,937 (25.6)	105 (94.3)	742 (57.8)	3 (100.0)	0
2022	Jul	4,564 (36.1)	6,634 (37.3)	8,718 (85.1)	680 (55.7)	1,926 (98.2)	478 (100.0)
2022	Aug	8,026 (56.7)	9,545 (66.8)	28,592 (76.3)	558 (53.6)	7,305 (97.8)	2,979 (99.8)
2022	Sep	4,948 (44.1)	4,069 (67.9)	10,630 (62.8)	320 (53.4)	3,415 (95.1)	1,289 (99.3)
2022	Oct	4,028 (21.9)	2,150 (45.5)	3,442 (58.0)	339 (54.6)	792 (88.4)	242 (98.3)
2022	Nov	3,467 (20.6)	1,569 (34.1)	1,561 (51.2)	275 (57.8)	302 (80.5)	94 (90.4)
Total		50,451 (32.1)	42,293 (39.6)	53,063 (73.2)	4,906 (51.8)	13,744 (96.3)	5,082 (99.5)

*Data from Florida immunization registry (FLSHOTS) for adults aged 18 y or older. Only the first dose of vaccine was used to determine the time the person was vaccinated. Percent in each column indicates the proportion of persons vaccinated by a county health department (CHD) provider. FDA, Food and Drug Administration.

†Persons receiving at least 1 dose of any FDA-approved vaccine against hepatitis A virus

‡Persons receiving at least 1 dose of any FDA-approved meningococcal vaccine serogroup ACWY (Menveo [GlaxoSmithKline, https://www.gsk.com], Menactra [Sanofi Pasteur, Inc., https://www.sanofi.us], MenQuadfi [Sanofi Pasteur, Inc.]).

§Persons receiving at least 1 dose of FDA emergency use authorized mpox vaccine JYNNEOS (Bavarian Nordic, https://www.bavarian-nordic.com).

¶Persons receiving, on the same day, at least 1 dose of both an FDA-approved vaccine against hepatitis A virus and 1 dose of an FDA-approved serogroup ACWY meningococcal vaccine. Persons enumerated in this column are also enumerated in columns for hepatitis A and meningococcal disease.

#Persons receiving, on the same day, at least 1 dose of JYNNEOS vaccine and either a dose of hepatitis A vaccine or serogroup ACWY meningococcal vaccine. Persons enumerated in this column are also enumerated, as appropriate, in columns for hepatitis A, meningococcal disease, and mpox.

**Persons receiving, on the same day, a dose of JYNNEOS vaccine, hepatitis A vaccine, and serogroup ACWY meningococcal vaccine. Persons enumerated in this column are also enumerated in columns for hepatitis A, meningococcal disease, and mpox.
